# The significance of macrophage phenotype in cancer and biomaterials

**DOI:** 10.1186/s40169-014-0041-2

**Published:** 2014-11-25

**Authors:** Hannah C Bygd, Kiva D Forsmark, Kaitlin M Bratlie

**Affiliations:** Department of Materials Science & Engineering, Iowa State University, Ames, 50011 Iowa USA; Department of Chemical & Biological Engineering, Iowa State University, Ames, 50011 Iowa USA; Ames National Laboratory, Ames, 50011 Iowa USA

**Keywords:** Macrophage reprogramming, Cancer, Biomaterials, Anti-angiogenic therapy, Recruitment inhibition, Tissue engineering, Foreign body response

## Abstract

Macrophages have long been known to exhibit heterogeneous and plastic phenotypes. They show functional diversity with roles in homeostasis, tissue repair, immunity and disease. There exists a spectrum of macrophage phenotypes with varied effector functions, molecular determinants, cytokine and chemokine profiles, as well as receptor expression. In tumor microenvironments, the subset of macrophages known as tumor-associated macrophages generates byproducts that enhance tumor growth and angiogenesis, making them attractive targets for anti-cancer therapeutics. With respect to wound healing and the foreign body response, there is a necessity for balance between pro-inflammatory, wound healing, and regulatory macrophages in order to achieve successful implantation of a scaffold for tissue engineering. In this review, we discuss the multitude of ways macrophages are known to be important in cancer therapies and implanted biomaterials.

**Electronic supplementary material:**

The online version of this article (doi:10.1186/s40169-014-0041-2) contains supplementary material, which is available to authorized users.

## Introduction

### Heterogeneity of Macrophages

Macrophages are considered to be functionally heterogeneous cells with different phenotypes representing distinct sublineages [1],[2]. The heterogeneity of these cells is attributed to their location in the tissue, due to microenvironmental signals that control the functional phenotype [1],[3]-[5]. In the presence of specific microenviromental signals, macrophages are able to switch from one phenotype to another, indicating that these cells have a degree of plasticity in addition to heterogeneity [3],[6]. In general, heterogeneity of macrophages can be described as a spectrum of phenotypes [1]-[3],[6]-[10]. One end represents classical macrophages activated with interferon (IFN)-γ, M(IFN-γ), and at the other end alternative macrophages activated by interleukin (IL)-4, M(IL-4) [7],[8],[11]-[15]. This new nomenclature, recently proposed by Murray et al., more accurately reflects the individual phenotypes and polarizations of these cells. Other variations of macrophages that lie along this spectrum include: M(Ic), activated by immune complexes (Ic); M(IL-10); those stimulated by glucocorticoids (GC) and transforming growth factor (TGF)-β, M(GC + TGF-β); M(GC); M(LPS), activated by lipopolysaccharides; and M(LPS + IFN-γ) [3],[6],[8]-[10]. Each of these phenotypes varies in their effector functions, molecular determinants, cytokine and chemokine profiles, as well as receptor expression.

Overall, classically activated, formerly referred to as M1, macrophages are known to be pro-inflammatory and cytotoxic. Macrophages are skewed towards this phenotype when IFNs and toll-like receptor (TLR) signaling activate IFN regulatory factor/signal transducers and activators of transcription (IRF/STAT) signaling pathways via STAT1 [7],[10],[15]-[18]. This transcription factor then causes macrophages to upregulate IRF5, which is essential for production of large amounts of pro-inflammatory cytokines [16], including tumor necrosis factor (TNF)-α, IL-1β, IL-1, IL-6, IL-8, IL-12, IL-15, IL-18, and IL-23 that elicit both T-helper (Th)1 and Th17 responses [9],[16],[18]-[20]. TLR stimulation can also activate nuclear factor (NF)-κB, such that p65/p50 heterodimers are formed and lead to the production of hypoxia-inducible factor (HIF)-1α [15],[21],[22]. This protein, found in the presence of low oxygen concentrations, regulates the NOS2 gene to increase the secretion of inducible nitric oxide synthase (iNOS) [21], toxic nitric oxide (NO), and reactive oxygen intermediates (ROI) [19]. A chemokine profile for classically activated macrophages may include HCC-2 (CCL15), macrophage inflammatory protein (MIP)-3α (CCL20), and B cell attracting chemokine-1 (CXCL13), as well as IFN-γ-inducible chemokines such as, monocyte chemotactic protein (MCP)-1 (CCL2), interferon-inducible T cell alpha chemoattractant (I-TAC) (CXCL11), interferon gamma-induced protein 10 (IP-10) (CXCL10) and monokine induced by gamma interferon (MIG) (CXCL9) [7],[18],[20]-[22]. Production of these chemokines can be a result of previously mentioned transcription factors STAT1 or NF- κB [16],[18]. These chemokines also coordinate natural killer (NK) and Th1 cell responses, integrating classically activated macrophages into the amplification and regulation of polarized T cell responses [20],[21]. Surface molecules expressed by classically activated macrophages include elevated amounts of MHC class II receptors; costimulatory molecules CD80 and CD86; IL-2Ra, IL-15Ra and IL-7R; and low levels of mannose receptor C type 1 (MRC1) and Fcγ RII [17],[18],[20]. Each of these characteristics allow classically activated macrophages to be potent effector cells that mediate resistance against bacterial, viral, and fungal infections as well as tumor cells [18],[19]. They are also important in the inflammatory stages of wound healing and the foreign body response (FBR) to biomaterials [23]-[25].

Alternatively activated, previously known as M2 macrophages, are said to be pro-angiogenic, promoting tissue remodeling and repair. This phenotype arises when IL-4 activates the IRF/STAT signaling pathway via STAT6 [7],[10],[15]-[18]. IL-10, on the other hand, activates STAT3-mediated alternative activation and gene expression [7],[15]-[18]. This STAT-mediated activation of macrophages is regulated by the suppressor of cytokine signaling (SOCS) family: where IL-4 can upregulate SOCS1, inhibiting the action of STAT1, but IFN-γ and TLR stimulation cause SOCS3 to be upregulated to prevent the activity of STAT3 [16],[26]. The transcription factors STAT3 and STAT6 allow for high-level production of the cytokines IL-10, IL-1 receptor antagonist (IL-1Rα), IL-4Rα, TGF-β, and the type II IL-1 decoy receptor [16],[18],[20],[21]. Other genes activated by STAT6 include mannose receptor (*Mrc1*), resistin-like α (*Retnla/Fizz1*), and chitinase 3-like 3 (*Chi3l3/Ym1*). For STAT3, some of the genes expressed are *Il10, Tgfb1,* and *Mrc1*[16]. STAT6 also coordinates with peroxisome proliferator-activated receptors PPARγ and PPARδ, as well as Krüppel-like factor (KLF)-4, to induce some alternative genes (*Arg-1, Mrc1, Fizz1, PPARγ*) while inhibiting genes associated with classical activation (*TNFa, Cox-2, CCL5, iNOS*) by preventing NF-κB activation [16]. However, NF-κB activation and the formation of p50 homodimers are also important in alternative activation and resolution of inflammation [15],[21],[22]. Chemokines induced by IL-4 or IL-13 alternative activation include monocyte chemotactic protein (MCP)-4 (CCL13), MCP-2 (CCL8), MCP-1 (CCL2), macrophage-derived chemokine (MDC) (CCL22), alternative macrophage activation-associated chemokine (AMAC)-1 (CCL18) and eotaxin-3 (CCL26) [7],[18],[20]-[22]. CCL22 specifically attracts Th2 and Treg cells, showing that alternative macrophages are also involved in the polarization of T cell responses [21]. Macrophages activated by IL-10, TGF-β, and GC produce the chemokines eotaxin-2 (CCL24), IP-10 (CXCL10), I-TAC (CXCL11), and regulated on activation, normal T cell expressed and secreted (RANTES) (CCL5) [20],[21]. Other factors produced include vascular endothelial growth factor (VEGF), matrix metalloproteinases (MMPs); and HIF-2α to regulate ARG1 and the arginase pathway to produce ornithine and polyamines [18]. The exception to alternative activation is the phenotype of macrophages induced by Ic; they retain the ability to produce high levels of pro-inflammatory cytokines [18]. Overall, alternatively activated macrophages are efficient phagocytic cells with the expression of mannose and galactose receptors; CD163, TLR8, TLR1, and IL21a; and MRC1 and scavenger receptor type 1 (SR-A1) [17],[18],[20]. They are involved in parasite containment, tumor progression, and function to dampen immune responses [12]. In the resolution stages of the FBR, alternative macrophages drive the wound healing response, often leading to fibrotic encapsulation and failure of implanted devices and scaffolds [23],[25].

## Review

### Macrophages as cancer therapeutic targets

#### Tumor-associated macrophages

Tumor-associated macrophages (TAMs) have properties consistent with alternatively activated macrophages [27]. They produce cytokines like IL-10 and TGF-β [21]. The polarization of macrophages recruited to a tumor site, or any other tissue, is highly dependent on the cytokines present. The production of both IL-10 and TGF-β suppresses anti-tumor activities of the immune system allowing tumor cells to avoid destruction by immune cells [28]. TAMs have been found to be poor producers of NO and ROIs, which are typically products of classically activated macrophages [29]. In addition, TAMs express low levels of cytokines such as IL-12, TNF-α, and IL-6 [29]. Lastly, TAMs have been found to be poor antigen-presenting cells indicating that they do not have the potent effector cell functions attributed to classically activated macrophages [19]. This information establishes that TAMs represent a subset of alternatively activated macrophages, and that many of their byproducts enhance tumor growth and angiogenesis.

While angiogenesis plays a central role in the progression of tumors from benign to malignant, there are many other factors involved. MMPs contribute to tumor invasion through matrix remodeling where they are capable of cleaving extracellular matrix (ECM) proteins [29], which normally provide a barrier for tumor growth. These MMPs along with other proteases such as plasmin and urokinase-type plasminogen activator (uPA) are all produced by TAMs [21],[29]. The continued proliferation and growth aided by TAMs can lead to metastasis of tumor cells. In metastasis, it is suggested that primary tumors are able to release factors that increase a metastatic outcome at other sites. These sites are referred to as premetastatic niches where the factors secreted by primary tumors cause the accumulation of myeloid progenitor cells [30]. A recent study has shown that TAMs play an important role in controlling the survival, migration and growth of metastatic cells to these niches [31]. TAMs were also found to enhance tumor cell extravasation, establishment and subsequent growth in surrounding tissue. The involvement of TAMs in tumor angiogenesis, growth, progression and metastasis makes them attractive targets for anti-cancer therapeutics. Therapeutic strategies directed at TAMs fall into four categories: reduction of effector function, limiting recruitment, prevention of pro-tumor polarization, and macrophage reprogramming [32],[33]; the benefits and drawbacks of which are outlined in Table 1[33]-[42].

**Table 1:** **Advantages and disadvantages of anti-cancer therapies targeting macrophage behaviors**

Approach	Advantages	Disadvantages
Anti-angiogenic therapy	Inhibit tumor growth and prevent metastasis [33],[34], improves efficacy of chemotherapeutics [35]	Must be used in combination with chemotherapeutics [36]; systemic effects [36],[37]
Recruitment inhibition	Prevent macrophages from entering tumor, becoming TAMs [38],[39]	Systemic effects [38]
Macrophage reprogramming	Macrophages secrete tumoricidal molecules [40],[41]	Local delivery necessary to avoid altering systemic Th1/Th2 paradigm [42]

### Anti-angiogenic therapy

Angiogenesis must occur in tumors for them to grow even small amounts [35]. This process can be influenced via a multitude of factors that are induced in hypoxic regions including VEGF, placental growth factor (PlGF), angiopoietins (ANGs), colony stimulating factor (CSF)-1, and CCL2/MCP-1 [35]. Anti-angiogenic therapy via the VEGF pathway, the primary angiogenic pathway of macrophages, is ineffective, as tumor cells are able to activate other pro-angiogenic pathways [36]. However, macrophage angiogenic abilities can be indirectly prohibited through the use of other factors. When a tumor develops regions of inadequate oxygen supply, HIF1-α subunits are stabilized, recruiting bone marrow (BM)-derived cells including macrophages that up regulate angiogenesis. The elimination of HIF1-α from the tumor environment provides a potential anti-angiogenic cancer therapy pathway by inhibiting the recruitment of macrophages and other pro-angiogenic cells [43]. HIF1-α knockout mice (HIFko) with glioblastoma (GBM) tumors, show a decrease in angiogenesis when compared to HIF functional mice with tumors [43].

**Figure 1:**
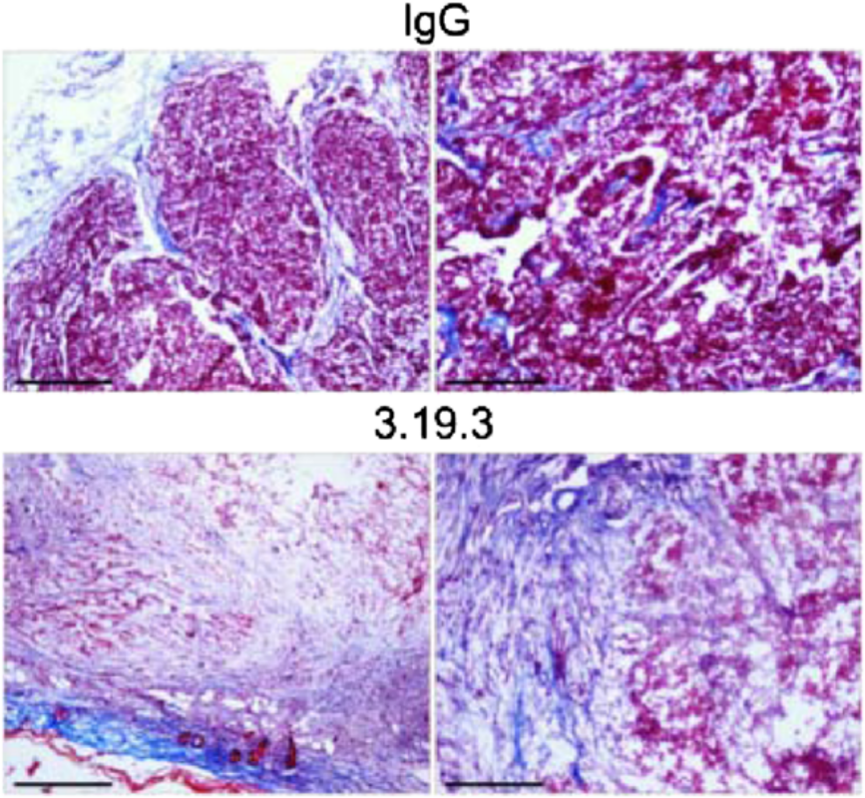
**Masson’s trichrome staining of orthotopic, late-stage MMTV-PyMT mammary tumors treated according to an extended (9 weeks) treatment schedule.** Collagen’s blue staining demonstrates abundant fibrotic tissue and scant tumor cells in 3.19.3-treated tumors (day 78). Left panels show tumor periphery. Scale bars, 600 mm (**left panels**) and 300 mm (**right panels**). Images are representative of five 3.19.3-treated (day 78) and three control IgG-treated (day 48) tumors. Reproduced with permission [45].

ANG2 is produced by endothelial cells in hypoxic environments and would typically recruit pro-angiogenic cells, however binding of ANG2 with a monoclonal antibody inhibited angiogenesis by blocking the interaction of ANG2 with TIE2-expressing monocytes [44],[45]. TIE2-expressing monocytes are a subpopulation of TAMs that have the greatest role in tumor angiogenesis [44]; preventing activation of these cells can halt their angiogenic activity and disable further recruitment of pro-angiogenic cells. Blocking of ANG2 with a monoclonal antibody inhibits tumor growth; causes regression of tumor vasculature by inducing apoptosis in some pro-angiogenic cells; and hinders progression of some late stage cancers (Figure 1) [45]. While the anti-angiogenic treatments mentioned here have not been shown to be extremely efficient alone, they may be used in combination with other chemotherapeutics to improve the outlook for patients [34],[37],[42].

### Recruitment inhibition

Another option for targeting TAMs is to inhibit the recruitment of monocytes to the primary tumor site [38],[39]. CXCL12 is a chemokine that is thought to regulate the migration of BM-derived cells, facilitating their transmigration through endothelial cell barriers into the tumor microenvironment [46]. Also, secretion of CXCL12 by stromal cells outside of the tumor microenvironment attracts cancer cells via their upregulated CXCR4 receptor [46]. Thus, several CXCR4 antagonists are being studied as additive cancer therapeutics to reduce tumor infiltration by BM-derived cells and prevent further metastatic spread [38]. One antagonist of interest is CTCE-9908, which is a chemokine-based therapy [47]-[49]. In prostate cancer cell lines (PC-3-Neo and PC-3-Bcl-2 transfected with *Bcl*-2), treatment with CTCE-9908 reduces VEGFR1and CD11b expressing cells [49]. Both VEGFR1 and CD11b are expressed on tumor-infiltrating cells that promote angiogenesis [15],[35],[36],[50]. Phase II clinical trials in hepatocellular carcinoma using CTCE-9908 have also been initiated [51].

**Figure 2:**
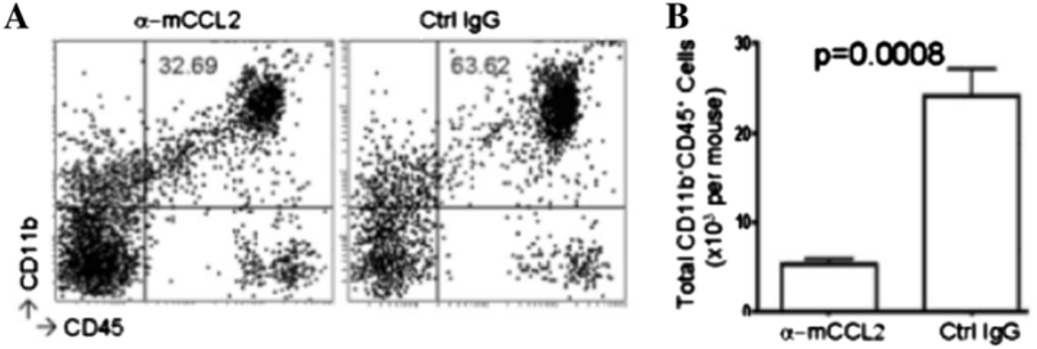
**C57BL/6 mice bearing GL261 glioma received 2 mg/kg/dose (approximately 40 μg/mouse) anti-mouse CCL2 mAb or control IgG twice weekly by i.p. injections starting on day 7 after tumor cell inoculation (n = 5/group).** On day 24, mice were euthanized and isolated BILs were pooled from all mice in the same treatment group, and evaluated by flow cytometry for surface expression of CD11b and CD45 **(A).** Absolute numbers of CD11b + CD45 + (p = 0.0008) **(B).** Reproduced with permission [53].

CCL2 is a chemokine that has been heavily investigated in prostate, ovarian and breast cancers because CCL2 regulates the recruitment of monocytes and macrophages to tumors and other sites of inflammation [38],[52]. In recent glioma therapy studies, a mAB-based CCL2 blockade reduced the percentage of CD11b^+^CD45^+^ TAMs by about 50% and decreased the total number of these cells five-fold (Figure 2) [53]. In a previous study, the use of anti-CCL2 decreased the overall burden of prostate tumors *in vivo* by 96% after 5 weeks [54]. Combining this therapy with the already in use, anti-mitotic chemotherapy medication, Docetaxel, further improved the results [54]. Since then, more work has been done to examine the synergy of these two treatments in preventing metastasis of primary prostate cancer to bone [55],[56].

As CD11b is a macrophage receptor that is important in recruitment to tumor sites, a CD11b antibody provides another treatment option for TAM targeted cancer therapy [50]. The use of a monoclonal CD11b antibody both enhances tumor response to radiation and reduces infiltration of myeloid cells [50]. Based on these examples, the targeting of chemokines and chemokine receptors has resulted in an effective enhancement of anti-cancer therapies by showing both decreased tumor size and prevention of tumor metastasis [38],[39].

### Macrophage reprogramming

Macrophage plasticity has led to the idea of utilizing macrophage reprogramming to synergistically act with chemotherapeutics. Many of the ways in which TAMs contribute to tumor development and survival are specific to the alternatively activated phenotype. Thus, being able to prevent TAMs from alternatively differentiating or promoting reprogramming of TAMs to classical macrophages will prevent tumor growth.

Several mechanisms of M2 macrophage polarization have been studied, and these pathways may also prove to be viable targets in cancer therapeutics. Jumonji domain containing-3 (Jmjd3) is a histone 3 Lys27 (H3k27) demethylase that has been implicated in regulating M2 macrophage polarization [57]. A deficiency of Jmjd3 results in trimethylation of H3k27 on the gene *Irf4*, which encodes a key transcription factor M2 activation [57]. Reactive oxygen species (ROS) production has also been found to play a critical role in macrophage differentiation [58]. Specifically, inhibition of superoxide (O^2−^) production prevents M2 macrophage polarization but does not hinder the M1 phenotype [58]. Thus, blocking of the Jmjd3-*Irf4* axis or ROS production may be potentially effective methods for added tumor inhibition.

**Figure 3:**
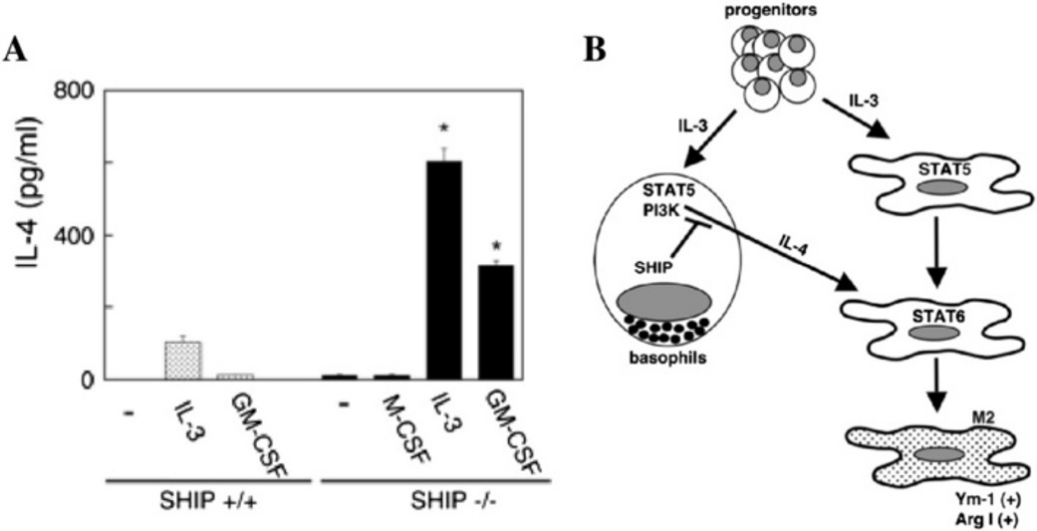
**Repressing IL-3-induced M2 macrophages through inhibiting IL-4 production from basophils. (A)** IL-3 and GM-CSF stimulate the production of more IL-4 from SHIP−/− than SHIP+/+ Lin- BM cells. SHIP+/+ and SHIP−/− Lin- BM cells were cultured with M-CSF, IL-3, or GM-CSF for 24 h and supernatants were subjected to IL-4 ELISAs. Data shown are the means ± SEM of duplicate determinations. *, p < 0.05 compared with unstimulated cells. **(B)** Model of IL-3-induced M2 skewing and the role that SHIP plays in this process. IL-3 stimulates the proliferation and differentiation of both basophil precursors and monocyte/macrophage progenitors. IL-3 also stimulates the production of IL-4 from basophils and basophil progenitors in a STAT5-dependent manner. SHIP within the basophils represses this IL-4 production. The secreted IL-4, in turn, skews, via STAT6, the maturing and mature MΦs to an M2 phenotype. Copyright 2009. The American Association of Immunologists, Inc. [59].

The differentiation of infiltrating monocytes into TAMs also results from cytokines like IL-4, IL-10, and IL-13. The use of IL-3 has been successful at inhibiting IL-4 produced by basophils, resulting in macrophages skewed towards a classical polarization [59]. SHIP (src-homology 2-containing inositol 5' phosphatase) is a molecule that negatively regulates the phosphatidylinositol-4,5-bisphosphate 3-kinase (PI3K) apoptotic pathway. In cancers, the PI3K pathway is overactive, allowing malignant cells to avoid apoptosis, essentially becoming immortal. It has been determined that basophils produce SHIP in response to IL-3, which can then inhibit IL-4 production necessary for TAM activation (Figure 3) [59].

Many tumor-infiltrating monocytes are alternatively activated by cytokines released by existing tumor cells [41]. However, the added presence of classical activators such as CpG oligodeoxynucleotides (CpG) and an anti-CD40 agonist can increase anti-tumor activity of macrophages. CpG causes a pro-inflammatory response in macrophages and the agonistic anti-CD40 can reverse immune suppression. As a follow up study to those that indicated that the synergistic effects of anti-CD40 and CpG increase classical activation [40], a combination of anti-CD40, CpG, and the chemotherapeutic agent cyclophosphamide was used to study treatment of melanoma *in vivo*. In this combinatorial study, there was an approximate ten-fold decrease in tumor size and survival was extended by ~12 days [60]. There was also an increase in the percentage of F4/80^+^Gr1^+^ inflammatory monocytes [60],[61].

Reprogramming of existing TAMs to be classical macrophages is another valid approach to improve upon conventional anticancer therapies. IFN-α has long been known to be tumoricidal and was the first cytokine to show some benefit in the treatment of some cancer types [62]. Because of the protein’s short half-life, however, the dose required for efficacy becomes toxic to healthy tissue and the tumor is only exposed to short bursts of therapy [62]. This is why the use of TIE2-expressing monocytes, which are regularly recruited to tumor sites, to selectively deliver IFN-α, can inhibit angiogenesis and skew macrophage polarization to the classical end of the spectrum [62]. This is shown by the presence of cells expressing Iba1, a monocyte/macrophage/microglia protein, in and around the tumor site.

Histidine-rich glycoprotein (HRG), a host produced anti-angiogenic and immunomodulatory factor to promote TAM reprogramming is another viable target [63]. HRG has been studied to identify mechanisms by which it mediates anti-tumor effects; and the results revealed that TAMs activated by HRG down regulated expression of pro-angiogenic cytokines and upregulated that of angiostatic cytokines. At the same time, HRG activated TAMs showed improved quality of existing vasculature causing an increase in the effectiveness of other chemotherapeutics [63]. Another target for reprogramming TAMS is the NF-κB signaling pathway [64]. Inhibition of NF-κB signaling was found with IκB kinase (IKK)β reduction, stimulating TAMs to become cytotoxic through recruitment of NK cells with the production of IL-12 [64]. These three examples, along with many more, provide proof-of-concept data for the reprogramming of macrophages in cancer therapeutics.

### Macrophages and scaffolds for tissue engineering

Macrophages are involved in ECM remodeling, proliferation of epithelial cells, development of vasculature and the organization of tissues during development [65]. These functional capacities of macrophages extend into the wound healing response and the FBR to biomaterials. Macrophage phenotype is dynamic throughout the course of these processes, and the balance between phenotypes is instrumental in the timely progression of these responses from injury to successful healing. As with TAMs, macrophages involved in healing retain their plasticity and alter their phenotype in response to a variable cytokine microenvironment in the progression of these processes [6].

### Overview of the foreign body response to implanted scaffolds

Surgical implantation or injection of a biomaterial-based construct injures the tissue, resulting in an influx of blood and cell death. Dying cells release danger signals (danger associated molecular patterns, DAMPs) that induce local inflammation [66] and activate resident macrophages [67],[68]. These DAMPs include HMGB1, histones, and uric acid [66],[67],[69],[70]. Blood proteins such as albumin, fibrinogen, fibronectin, immunoglobulin G (IgG), and various complement proteins adsorb to the surface of the biomaterial [71]. Activation of the complement cascade results in opsonization of the biomaterial surface with C3b and induces inflammation through the anaphylatoxins C3a and C5a [72]. These anaphylatoxins recruit leukocytes to the site of inflammation, cause histamine release from mast cells, and induce oxidative bursts in neutrophils [73]. Release of histamine from mast cells attracts neutrophils and monocytes [74],[75]. Neutrophils are the first immune cells to arrive at the implant site [76] and, along with mast cells, secrete IL-4 and IL-13 early in innate immune responses [9].

Monocytes are the next immune cells to extravasate into the tissue where they differentiate into tissue macrophages [77]. These macrophages are classically activated upon the adsorbed protein layer [78],[79]. Proteins, such as fibrinogen, C3, and C3b on the surface of the biomaterial are bound by the integrin αMβ2 (CD11b:CD18), also known as complement receptor 3 (CR3), on the surface of macrophages [77],[80]-[82]. Activated macrophages secrete TNF-α, IL-6, IL-8, MCP-1, RANTES, ROS, iNOS, IL-1β, and MMPs [83]-[85]. The chemokines MIP-1α, IL-8, and MCP-1 attract additional monocytes [83]. These biomaterial-activated macrophages are also characterized by an increased phagocytic capacity [86]. Continued presence of pro-inflammatory macrophages causes acute inflammation to morph into chronic inflammation [87].

Attempted phagocytosis of biomaterials leads to the fusion of adherent classically activated macrophages into foreign body giant cells (FBGCs) [88]. IL-4 and IL-13 induce the fusion of adherent macrophages [88]. β1 and β2 integrins are involved in the fusion of these macrophages [89], and CCL2 guides the chemotaxis of adherent macrophages towards each other [90]. FBGCs have a cytokine profile more characteristic of alternatively activated macrophages that includes TGF-β, platelet derived growth factor (PDGF), IL-1rα, and IL-10 [9],[77],[84],[91]. FBGCs secrete protons, ROS, and MMPs in an attempt to eradicate the foreign body [92],[93]. Like M1 macrophages, FBGCs secrete pro-inflammatory RANTES and the chemoattractant MCP-1 [84]. ECM breakdown by MMPs leads to increased DAMPs in the microenvironment and further macrophage activation [94].

The resolution stage of the FBR is dominated by alternatively activated macrophages. A profibrotic, alternatively activated, wound healing macrophage phenotype results from macrophage phagocytosis of dying cells, stimulation by IL-4 or by IL-13 [12],[95]. These dying cells include epithelial and endothelial cells that are damaged by pro-inflammatory cytokines, such as TNF-α, and short-lived neutrophils [25],[96]. Alternatively activated macrophages secrete profibrotic mediators such as TGF-β, IL-4, IL-13, IL-10, arginase, and ECM components [9],[97]. These macrophages drive the wound healing response by activating mesenchymal cells that participate in the wound healing process [98],[99]. TGF-β can also induce an M2-like phenotype in macrophages [100]. These M2 macrophages are profibrotic, but are still unable to reduce the pro-inflammatory response. Reduction of chronic inflammation requires IL-10-induced activation of regulatory M2-like macrophages [9],[101]. These macrophages secrete high levels of the same protein that activates them [9]. IL-10 prevents the translation of pro-inflammatory cytokines by macrophages through STAT3 [102],[103].

As in the immune response to parasitic infections, the early phase of wound healing and the FBR is characterized by M1-like macrophages and the late phases are controlled by M2-like macrophages [25],[91],[104]-[106]. In the healing of aseptic wounds regulatory M2 (IL-10 stimulated) macrophages rapidly downregulate the inflammatory response to promote tissue repair [9],[107]-[110]. Conversely, in the FBR, further activation of macrophages will occur, resulting in continued chronic inflammation (pro-inflammatory macrophages and FBGCs) and continued wound healing (wound healing macrophages).

**Figure 4:**
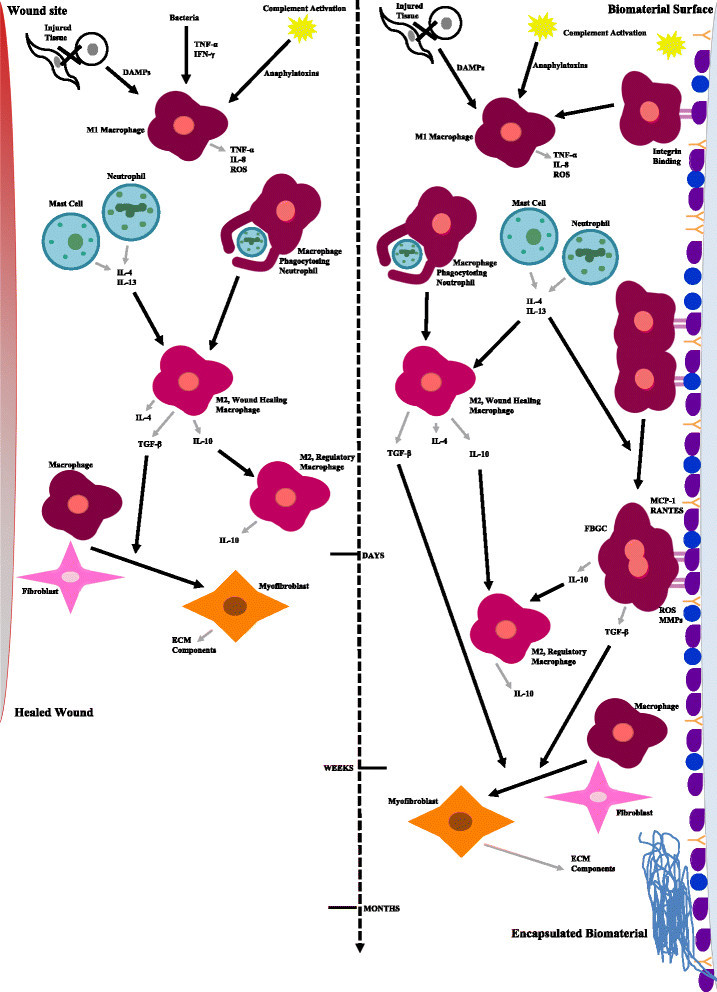
**Macrophage phenotype in the wound healing and foreign body responses.**

It has long been hypothesized that chronic inflammation is present until an extensive fibrous capsule surrounds the biomaterial [76]. Resident fibroblasts, fibrocytes, and macrophages are activated by TGF-β, and become myofibroblasts [111]-[115]. Myofibroblasts secrete high amounts of collagen I, collagen III, and fibronectin [110],[116]. The expression of α-smooth muscle actin (α-SMA) permits myofibroblasts to contract collagen networks in a process known as contractile scarring [117],[118]. Incessant activation of myofibroblasts results in continued secretion and contraction of ECM components. This eventually results in excessive scarring, and fibrous encapsulation. The fibrous capsule is a dense, hypocellular, avascular collagenous network that reduces the diffusion of all molecules, and results in the failure of scaffolds for applications in tissue engineering [119],[120]. The entire process leading up to fibrous encapsulation is illustrated in Figure 4.

### Scaffolds to instruct phenotypic macrophage responses

Depending on biomaterial properties and the cytokines secreted by inflammatory cells in the biomaterial microenvironment, macrophages adopt either an M1- or M2-like state [23]. As macrophages are plastic, they can exist on a spectrum between these two states. This leads to the hypothesis that surface chemistry and physical properties of scaffolds can be used to polarize macrophages towards a specific phenotype, or away from another. In particular, some scaffolds have been engineered to reduce prolonged activation of M1-like macrophages, so that cell-laden scaffolds maintain cell viability [121],[122]. Additional scaffolds have been engineered to reduce excessive fibrosis and decrease time to incorporation of the implant [123]. A balance in macrophage phenotype must be achieved for scaffold vascularization.

Varied scaffold chemistries suggest the ability to decrease the expression of M1 macrophages. Microgel conformational coatings formed from poly(N-isopropylacrylamide) (pNIPAm) and poly(ethylene glycol) diacrylate (PEGDA) reduce fibrinogen adsorption, macrophage adhesion, macrophage spreading, and secretion of inflammatory cytokines [124]. Zwitterionic hydrogels are able to reduce protein adsorption and are characterized by anti-inflammatory, pro-healing macrophages that promote angiogenesis and show no evidence of a collagenous capsule for longer than three months [125]. The ability of macrophages to induce positive tissue remodeling on fourteen different biologically-derived surgical meshes was investigated, and suggested that a predominance of M2 macrophages could potentially lead to more constructive tissue remodeling after two weeks [23]. Sugisis and Matristem scaffolds – derived from porcine small intestinal submucosa, and urinary bladder, respectively – appeared to increase macrophage infiltration; whereas the other scaffolds, derived from human and porcine dermis, appeared to prolong the healing response and exhibited an increase in M1-like macrophages [23].

In addition to chemical properties, physical properties of scaffolds can significantly influence macrophage phenotype. Controlling the pore size of scaffolds is one technique that shows promise in decreasing pro-inflammatory macrophage presence and improving the healing outcome. A pore size of 30–40 μm within porous template scaffolds formed of five different synthetic polymers and one natural polymer appeared to increase infiltration of macrophages and vascular density, suggesting that these materials induce regenerative macrophages [126]-[128]. It is generally thought that geometric restriction of macrophages within these pores prevents them from spreading out into their phagocytic, inflammatory phenotype [24],[129],[130]. Vascular density is suggested to peak at pores size of 35 μm [126],[131]. The degree of porosity in a material can also influence macrophage phenotypes, with more porous materials leading to decreased healing time of implants and, therefore, a reduced fibrous capsule thickness. For example, even though porous polytetrafluoroethylene (PTFE) surfaces seemed to induce inflammatory cytokine secretion by macrophages, a thinner fibrous capsule was formed on porous versus nonporous PTFE [132]. BMDMs cultured on electrospun polydioxanone (PDO) of larger fiber length and pore size showed increased arginase, TGF-β, VEGF, and basic fibroblast growth factor expression, characteristic of alternatively activated macrophages, than those cells cultured on scaffolds with smaller fiber length and pore size [123].

Substrate morphology and surface topography represent two other physical properties of scaffolds that are thought to influence macrophage phenotype and thus the foreign body response and material biocompatibility. 2D and 3D sP(EO-*stat*-PO) surface modified poly(D,L-lactide-co-glycolide) (PLGA) substrates were compared to find that the flat surfaces studied in this work lead to pro-inflammatory cytokine profiles while 3D nanofibers resulted in increased pro-angiogenic chemokines and angiogenesis [133],[134]. Micro- and nano-structured surfaces have also been examined to determine the effect of surface topography on macrophage behavior [135]-[140]. Several studies have suggested macrophage responses are more greatly impacted by micro-patterned surfaces than corresponding nanostructures [135]-[137],[140], however, few distinctive correlations have been revealed. Some trends indicate that larger posts or widely separated posts on material surfaces induce anti-inflammatory phenotypes in macrophages [135],[136], while others suggest that nanostructured versus microstructured grooves decrease the pro-inflammatory response of macrophages [137],[138],[140]. Another surface property that has been examined with respect to macrophage phenotype is fiber diameter and orientation. Results from these studies indicate that aligned rather than randomly oriented nanofibers minimize inflammatory responses [121],[141].

The processing of biologic scaffolds appears to alter macrophage phenotype. Processing of scaffolds such as subintestinal submucosa with a carbodiimide crosslinker can lead to a predominately M1 response resulting in chronic inflammation and prolonged healing; whereas the non-crosslinked scaffold appeared to induce a large M2-like response and constructive remodeling at sixteen weeks [24]. A low degree of acetylated chitosan scaffolds (5%) is suggested to induce a macrophage response characteristic of M2 macrophages and a reduced fibrous capsule. However, the 15% degree of acetylation resulted in adherent, activated pro-inflammatory macrophages [122],[142], which again suggests that surface chemistry plays a role in macrophage response. Infiltration of blood vessels into a glutaraldehyde-crosslinked collagen scaffold was characterized by coordinated levels of M1- and M2-like macrophages [143].

It is suggested herein that a temporal balance between pro-inflammatory, wound healing, and regulatory (IL-10 stimulated) macrophages may be necessary for successful implantation of a scaffold for tissue engineering applications. Scaffold chemistry, pore size, and processing conditions appear to have the potential to regulate macrophage phenotype, and, therefore, the extent of inflammation, fibrous encapsulation, and angiogenesis of these materials. The effects of these biomaterial properties on macrophage phenotype are outlined in Table 2.

**Table 2:** **Biomaterial influence on macrophage phenotype**

Biomaterial property	Macrophage response
Large fibers and pores (PDO)	M2 response, wound healing, angiogenesis [123]
Fiber size	
~0.6 μm (PLLA)	Minimal M1 activation, low FBGC population [121]
~1.6 μm (PLLA)	High FBGC population [121]
Hydrogels with pores (30–40 μm) (pHEMA-co-MAA)	M2 dominated, maximum vascularization, minimum fibrotic response [132]
Microgel coating (pNIPAm-co-PEGDA)	Reduction of M1 activation and cytokine secretion [124]
Zwitterionic hydrogels	Anti-inflammatory, pro-healing M2 macrophages, angiogenesis, no fibrous capsule [125]
Subintestinal submucosa	
Crosslinked with carbodiimide	M1 bias, chronic inflammation, prolonged healing [24]
Non-crosslinked	M2 bias, constructive remodeling [24]
Acetylated chitosan	
5% acetylated	Predominately M2, reduced fibrous capsule [122],[142]
15% acetylated	Presence of M1 macrophages [122],[142]
Glutaraldehyde crosslinked collagen	M1/M2 balance, improved vascularization [143]
Biologically-derived scaffolds	
Porcine submucosa, urinary bladder	M2, timely constructive tissue remodeling [23]
Human, porcine dermis	M1, prolonged healing [23]

## Conclusions

Based on the information discussed here, it can be concluded that macrophages are an appealing and effective target for supplementing current cancer treatments. Thus far there is a lack of research leading to an understanding of how to achieve the appropriate balance of macrophage phenotypes at tumor and implant sites. In targeting macrophages with cancer therapeutics, the intention is to develop localized and target-specific treatment options. Several challenges exist and are outlined in Table 1[36]-[38],[42]. One such challenge lies in complete reprogramming to classically activated macrophages, which could yield a systemic loss of alternative macrophages resulting in hazardous levels of cytotoxic cytokines and significant amounts of tissue damage [9]. Classical macrophages are also necessary for basic immunological responses to infection, so an exclusively alternative macrophage population may leave patients susceptible to routine infections. Lastly, alternative macrophages are extremely important in wound healing and a deficiency may leave tissues unrepaired with no chance for recovery. Exploiting macrophages to co-opt tumors holds a number of advantages that could synergistically interact with existing chemotherapeutics. However, several challenges remain in reprogramming macrophages in the tumor microenvironment, including targeted delivery to the tumor site and selective delivery to alternatively activated TAM populations. Immunomodulation of macrophages may also result in improved success of implants for tissue engineering. In the FBR, the complete absence of M1 macrophages is detrimental in the progression of the response. Specifically for successful implantation of a scaffold for tissue engineering, M1 macrophages are necessary to instigate the inflammatory response and initiate the M2-coordinated wound healing process. Timely resolution of the response requires the presence of IL-10^high^ M2 macrophages. Though many of the materials mentioned here lead to a timely resolution of the FBR and successful vascularization, these materials cannot be used for all tissue engineering applications. Therefore, strategies to modulate macrophages in the tumor and biomaterial microenvironment require consideration of the desired end result.

## Authors’ original submitted files for images

Below are the links to the authors’ original submitted files for images. Authors’ original file for figure 1 Authors’ original file for figure 2 Authors’ original file for figure 3 Authors’ original file for figure 4 Authors’ original file for figure 5 Authors’ original file for figure 6

## Abbreviations

IFN: Interferon
IL: Interleukin
Ic: Immune complexes
GC: Glucocorticoids
TGF: Transforming growth factor
LPS: Lipopolysaccharides
M1: Classically activated, pro-inflammatory cytotoxic macrophages
TLR: Toll-like receptor
IRF: Interferon regulator factor
STAT: Signal transducers and activators of transcription
TNF: Tumor necrosis factor
Th: T-helper
NF: Nuclear factor
HIF: Hypoxia-inducible factor
iNOS: Inducible nitric oxide synthase
NO: Nitric oxide
ROI: Reactive oxygen intermediates
MIP: Macrophage inflammatory protein
MCP: Monocyte chemotactic protein
I-TAC: Interferon-inducible T cell alpha chemoattractant
IP: Interferon gamma-induced protein
MIG: Monokine induced by gamma interferon
NK: Natural killer
MRC1: Mannose receptor C type 1
FBR: Foreign body response
M2: Alternatively activated, pro-angiogenic, wound healing macrophages
SOCS: Suppressor of cytokine signaling
IL-1Rα: Interleukin-1 receptor agonist
*Mrc1*: Mannose receptor gene
*Retnla/Fizz1*: Resistin-like α gene
*Chi3l3/Ym1*: Chitinase 3-like 3 gene
PPARγ: Peroxisome proliferator-activated receptors
KLF: Krüppel-like factor
MDC: Macrophage-derived chemokine
AMAC: Alternative macrophage activation-associated chemokine
Treg: Regulatory T cell
VEGF: Vascular endothelial growth factor
MMPs: Matrix metalloproteinases
SR-A1: Scavenger receptor type 1
TAMs: Tumor-associated macrophages
ECM: Extracellular matrix
uPA: Urokinase-type plasminogen activator
PlGF: Placental growth factor
ANGs: Angiopoietins
CSF: Colony stimulating factor
BM: Bone marrow
HIFko: Hypoxia-inducible factor-α knockout mice
GBM: Glioblastoma
TIE: Tyrosine kinase with immunoglobulin-like and EGF-like domains
mAB: Monoclonal antibody
i.p.: Intraperitoneal
Jmjd3: Jumonji domain containing-3
H3k27: Histone 3 Lys27
ROS: Reactive oxygen species
SHIP: Src-homology 2-containing inositol 5’ phosphatase
PI3K: Phosphatidylinositol-4,5-bisphosphate 3-kinase
GM-CSF: Granulocyte macrophage colony-stimulating factor
SEM: Standard error of the mean
CpG: CpG oligodeoxynucleotides
HRG: Histidine-rich glycoprotein
IKK: IκB kinase
DAMPs: Danger associated molecular patterns
HMGB1: High-mobility group protein B1
IgG: Immunoglobulin G
C: Complement protein
CR3: Complement receptor 3
ENA-78: CXCL5
FBGCs: Foreign body giant cells
PDGF: Platelet derived growth factor
α-SMA: α-smooth muscle actin
pNIPAm: Poly(N-isopropylacrylamide)
PEGDA: Poly(ethylene glycol) diacrylate
PTFE: Polytetrafluoroethylene
BMDMs: Bone-marrow derived macrophages
PDO: Polydioxanone
PLGA: Poly(D,L-lactide-co-glycolide
PLLA: Poly-L-lactic acid
pHEMA-co-MAA: Poly(2-hydroxyethyl methacrylate-co methyl methacrylate)
RANTES: Regulated on activation, normal T cell expressed and secreted

## References

[1] Stout RD, Watkins SK, Suttles J (2009). Functional plasticity of macrophages: in situ reprogramming of tumor-associated macrophages. J Leukoc Biol.

[2] Gordon S, Taylor PR (2005). Monocyte and macrophage heterogeneity. Nat Rev Immunol.

[3] Stout RD, Suttles J (2004). Functional plasticity of macrophages: reversible adaptation to changing microenvironments. J Leukoc Biol.

[4] Akilbekova D, Philiph R, Graham A, Bratlie KM (2014). Macrophage reprogramming: influence of latex beads with various functional groups on macrophage phenotype and phagocytic uptake in vitro. J Biomed Mater Res Part A.

[5] McWhorter FY, Wang T, Nguyen P, Chung T, Liu WF (2013). Modulation of macrophage phenotype by cell shape. Proc Natl Acad Sci U S A.

[6] Stout RD, Jiang C, Matta B, Tietzel I, Watkins SK, Suttles J (2005). Macrophages sequentially change their functional phenotype in response to changes in microenvironmental influences. J Immunol.

[7] Martinez FO, Gordon S (2014). The M1 and M2 paradigm of macrophage activation: time for reassessment. F1000Prime Rep.

[8] Murray PJ, Allen JE, Biswas SK, Fisher EA, Gilroy DW, Goerdt S, Gordon S, Hamilton JA, Ivashkiv LB, Lawrence T, Locati M, Mantovani A, Martinez FO, Mege J-L, Mosser DM, Natoli G, Saeij JP, Schultze JL, Shirey KA, Sica A, Suttles J, Udalova I, van Ginderachter JA, Vogel SN, Wynn TA (2014). Macrophage activation and polarization: nomenclature and experimental guidelines. Immunity.

[9] Mosser DM, Edwards JP (2008). Exploring the full spectrum of macrophage activation. Nat Rev Immunol.

[10] Xue J, Schmidt SV, Sander J, Draffehn A, Krebs W, Quester I, De Nardo D, Gohel TD, Emde M, Schmidleithner L, Ganesan H, Nino-Castro A, Mallmann MR, Labzin L, Theis H, Kraut M, Beyer M, Latz E, Freeman TC, Ulas T, Schultze JL (2014). Transcriptome-based network analysis reveals a spectrum model of human macrophage activation. Immunity.

[11] Gordon S, Martinez FO (2010). Alternative activation of macrophages: mechanism and functions. Immunity.

[12] Gordon S (2003). Alternative activation of macrophages. Nat Rev Immunol.

[13] Lawrence T, Natoli G (2011). Transcriptional regulation of macrophage polarization: enabling diversity with identity. Nat Rev Immunol.

[14] Taub DD, Cox GW (1995). Murine Th1 and Th2 cell clones differentially regulate macrophage nitric oxide production. J Leukoc Biol.

[15] Wynn TA, Chawla A, Pollard JW (2013). Macrophage biology in development, homeostasis and disease. Nature.

[16] Sica A, Mantovani A (2012). Macrophage plasticity and polarization : in vivo veritas. J Clin Invest.

[17] Wolfs IMJ, Donners MMPC, de Winther MPJ (2011). Differentiation factors and cytokines in the atherosclerotic plaque micro-environment as a trigger for macrophage polarisation. Thromb Haemost.

[18] Mantovani A, Sica A, Sozzani S, Allavena P, Vecchi A, Locati M (2004). The chemokine system in diverse forms of macrophage activation and polarization. Trends Immunol.

[19] Sica A, Schioppa T, Mantovani A, Allavena P (2006). Tumour-associated macrophages are a distinct M2 polarised population promoting tumour progression: potential targets of anti-cancer therapy. Eur J Cancer.

[20] Martinez FO, Sica A, Mantovani A, Locati M (2008). Macrophage activation and polarization. Front Biosci.

[21] Mantovani A, Sozzani S, Locati M, Allavena P, Sica A (2002). Macrophage polarization: tumor-associated macrophages as a paradigm for polarized M2 mononuclear phagocytes. Trends Immunol.

[22] Biswas SK, Chittezhath M, Shalova IN, Lim J-Y (2012). Macrophage polarization and plasticity in health and disease. Immunol Res.

[23] Brown BN, Londono R, Tottey S, Zhang L, Kukla KA, Wolf MT, Daly KA, Reing JE, Badylak SF (2012). Macrophage phenotype as a predictor of constructive remodeling following the implantation of biologically derived surgical mesh materials. Acta Biomater.

[24] Badylak SF, Valentin JE, Ravindra AK, McCabe GP, Stewart-Akers AM (2008). Macrophage phenotype as a determinant of biologic scaffold remodeling. Tissue Eng Part A.

[25] Mantovani A, Biswas SK, Galdiero MR, Sica A, Locati M (2013). Macrophage plasticity and polarization in tissue repair and remodelling. J Pathol.

[26] Mantovani A, Locati M (2013). Tumor-associated macrophages as a paradigm of macrophage plasticity, diversity, and polarization: lessons and open questions. Arterioscler Thromb Vasc Biol.

[27] Kawamura K, Komohara Y, Takaishi K, Katabuchi H, Takeya M (2009). Detection of M2 macrophages and colony-stimulating factor 1 expression in serous and mucinous ovarian epithelial tumors. Pathol Int.

[28] Solinas G, Germano G, Mantovani A, Allavena P (2009). Tumor-associated macrophages (TAM) as major players of the cancer-related inflammation. J Leukoc Biol.

[29] Allavena P, Sica A, Solinas G, Porta C, Mantovani A (2008). The inflammatory micro-environment in tumor progression: the role of tumor-associated macrophages. Crit Rev Oncol Hematol.

[30] Qian B-Z, Pollard JW (2010). Macrophage diversity enhances tumor progression and metastasis. Cell.

[31] Qian B, Deng Y, Im JH, Muschel RJ, Zou Y, Li J, Lang RA, Pollard JW (2009). A distinct macrophage population mediates metastatic breast cancer cell extravasation, establishment and growth. PLoS One.

[32] Ruffell B, Affara NI, Coussens LM (2012). Differential macrophage programming in the tumor microenvironment. Trends Immunol.

[33] De Palma M, Lewis CE (2013). Macrophage regulation of tumor responses to anticancer therapies. Cancer Cell.

[34] Zhang W, Zhu X-D, Sun H-C, Xiong Y-Q, Zhuang P-Y, Xu H-X, Kong L-Q, Wang L, Wu W-Z, Tang Z-Y (2010). Depletion of tumor-associated macrophages enhances the effect of sorafenib in metastatic liver cancer models by antimetastatic and antiangiogenic effects. Clin Cancer Res.

[35] Squadrito ML, De Palma M (2011). Macrophage regulation of tumor angiogenesis: implications for cancer therapy. Mol Aspects Med.

[36] Bergers G, Hanahan D (2008). Modes of resistance to anti-angiogenic therapy. Nat Rev Cancer.

[37] Jain RK (2005). Antiangiogenic therapy for cancer: current and emerging concepts. Oncology.

[38] Garber K (2009). First results for agents targeting cancer-related inflammation. J Natl Cancer Inst.

[39] Mantovani A, Sica A (2010). Macrophages, innate immunity and cancer: balance, tolerance, and diversity. Curr Opin Immunol.

[40] Buhtoiarov IN, Lum HD, Berke G, Sondel PM, Rakhmilevich AL (2005). Synergistic activation of macrophages via CD40 and TLR9 results in T cell independent antitumor effects. J Immunol.

[41] Guiducci C, Vicari AP, Sangaletti S, Trinchieri G (2005). Redirecting in vivo elicited tumor infiltrating macrophages and dendritic cells towards tumor rejection. Cancer Res.

[42] Zeisberger SM, Odermatt B, Marty C, Zehnder-Fjällman AHM, Ballmer-Hofer K, Schwendener RA (2006). Clodronate-liposome-mediated depletion of tumour-associated macrophages: a new and highly effective antiangiogenic therapy approach. Br J Cancer.

[43] Du R, Lu KV, Petritsch C, Liu P, Ganss R, Passegué E, Song H, Vandenberg S, Johnson RS, Werb Z, Bergers G (2008). HIF1alpha induces the recruitment of bone marrow-derived vascular modulatory cells to regulate tumor angiogenesis and invasion. Cancer Cell.

[44] Coffelt SB, Tal AO, Scholz A, De Palma M, Patel S, Urbich C, Biswas SK, Murdoch C, Plate KH, Reiss Y, Lewis CE (2010). Angiopoietin-2 regulates gene expression in TIE2-expressing monocytes and augments their inherent proangiogenic functions. Cancer Res.

[45] Mazzieri R, Pucci F, Moi D, Zonari E, Ranghetti A, Berti A, Politi LS, Gentner B, Brown JL, Naldini L, De Palma M (2011). Targeting the ANG2/TIE2 axis inhibits tumor growth and metastasis by impairing angiogenesis and disabling rebounds of proangiogenic myeloid cells. Cancer Cell.

[46] Sun X, Cheng G, Hao M, Zheng J, Zhou X, Zhang J, Taichman RS, Pienta KJ, Wang J (2010). CXCL12 / CXCR4 / CXCR7 chemokine axis and cancer progression. Cancer Metastasis Rev.

[47] Kim SY, Lee CH, Midura BV, Yeung C, Mendoza A, Hong SH, Ren L, Wong D, Korz W, Merzouk A, Salari H, Zhang H, Hwang ST, Khanna C, Helman LJ (2008). Inhibition of the CXCR4/CXCL12 chemokine pathway reduces the development of murine pulmonary metastases. Clin Exp Metastasis.

[48] Richert MM, Vaidya KS, Mills CN, Wong D, Korz W, Hurst DR, Welch DR (2009). Inhibition of CXCR4 by CTCE-9908 inhibits breast cancer metastasis to lung and bone. Oncol Rep.

[49] Porvasnik S, Sakamoto N, Kusmartsev S, Eruslanov E, Kim W-J, Cao W, Urbanek C, Wong D, Goodison S, Rosser CJ (2009). Effects of CXCR4 antagonist CTCE-9908 on prostate tumor growth. Prostate.

[50] Ahn G-O, Tseng D, Liao C-H, Dorie MJ, Czechowicz A, Brown JM (2010). Inhibition of Mac-1 (CD11b/CD18) enhances tumor response to radiation by reducing myeloid cell recruitment. Proc Natl Acad Sci U S A.

[51] Wong D, Korz W (2008). Translating an antagonist of chemokine receptor CXCR4: from bench to bedside. Clin Cancer Res.

[52] Zhang J, Patel L, Pienta KJ (2010). CC chemokine ligand 2 (CCL2) promotes prostate cancer tumorigenesis and metastasis. Cytokine Growth Factor Rev.

[53] Zhu X, Fujita M, Snyder LA, Okada H (2011). Systemic delivery of neutralizing antibody targeting CCL2 for glioma therapy. J Neurooncology.

[54] Loberg RD, Ying C, Craig M, Day LL, Sargent E, Neeley C, Wojno K, Snyder LA, Yan L, Pienta KJ (2007). Targeting CCL2 with systemic delivery of neutralizing antibodies induces prostate cancer tumor regression in vivo. Cancer Res.

[55] Rozel S, Galbán CJ, Nicolay K, Lee KC, Sud S, Neeley C, Snyder LA, Chenevert TL, Rehemtulla A, Ross BD, Pienta KJ (2009). Synergy between anti-CCL2 and docetaxel as determined by DW-MRI in a metastatic bone cancer model. J Cell Biochem.

[56] Kirk PS, Koreckij T, Nguyen HM, Brown LG, Snyder LA, Vessella RL, Corey E (2013). Inhibition of CCL2 Signaling in Combination with Docetaxel Treatment Has Profound Inhibitory Effects on Prostate Cancer Growth in Bone. Int J Mol Sci.

[57] Satoh T, Takeuchi O, Vandenbon A, Yasuda K, Tanaka Y, Kumagai Y, Miyake T, Matsushita K, Okazaki T, Saitoh T, Honma K, Matsuyama T, Yui K, Tsujimura T, Standley DM, Nakanishi K, Nakai K, Akira S (2010). The Jmjd3-Irf4 axis regulates M2 macrophage polarization and host responses against helminth infection. Nat Immunol.

[58] Zhang Y, Choksi S, Chen K, Pobezinskaya Y, Linnoila I, Liu Z-G (2013). ROS play a critical role in the differentiation of alternatively activated macrophages and the occurrence of tumor-associated macrophages. Cell Res.

[59] Kuroda E, Ho V, Ruschmann J, Antignano F, Hamilton M, Rauh MJ, Antov A, Flavell RA, Sly LM, Krystal G (2009). SHIP represses the generation of IL-3-induced M2 macrophages by inhibiting IL-4 production from basophils. J Immunol.

[60] Johnson EE, Buhtoiarov IN, Baldeshwiler MJ, Felder MA, Van Rooijen N, Sondel PM, Rakhmilevich AL (2011). Enhanced T cell-independent antitumor effect of cyclophospamide combined with anti-CD40 mAb and CpG in mice. J Immunother.

[61] Arora M, Poe SL, Ray A, Ray P (2011). LPS-induced CD11b + Gr1(int)F4/80+ regulatory myeloid cells suppress allergen-induced airway inflammation. Int Immunopharmacol.

[62] De Palma M, Mazzieri R, Politi LS, Pucci F, Zonari E, Sitia G, Mazzoleni S, Moi D, Venneri MA, Indraccolo S, Falini A, Guidotti LG, Galli R, Naldini L (2008). Tumor-targeted interferon-alpha delivery by Tie2-expressing monocytes inhibits tumor growth and metastasis. Cancer Cell.

[63] Rolny C, Mazzone M, Tugues S, Laoui D, Johansson I, Coulon C, Squadrito ML, Segura I, Li X, Knevels E, Costa S, Vinckier S, Dresselaer T, Åkerud P, De Mol M, Salomäki H, Phillipson M, Wyns S, Larsson E, Buysschaert I, Botling J, Himmelreich U, Van Ginderachter JA, De Palma M, Dewerchin M, Claesson-Welsh L, Carmeliet P (2011). HRG inhibits tumor growth and metastasis by inducing macrophage polarization and vessel normalization through downregulation of PlGF. Cancer Cell.

[64] Hagemann T, Lawrence T, McNeish I, Charles KA, Kulbe H, Thompson RG, Robinson SC, Balkwill FR (2008). “Re-educating” tumor-associated macrophages by targeting NF-kappaB. J Exp Med.

[65] Pollard JW (2009). Trophic macrophages in development and disease. Nat Rev Immunol.

[66] Klune JR, Dhupar R, Cardinal J, Billiar TR, Tsung A (2008). HMGB1: endogenous danger signaling. Mol Med.

[67] Bianchi ME (2007). DAMPs, PAMPs and alarmins: all we need to know about danger. J Leukoc Biol.

[68] Osterloh A, Kalinke U, Weiss S, Fleischer B, Breloer M (2007). Synergistic and differential modulation of immune responses by Hsp60 and lipopolysaccharide. J Biol Chem.

[69] Huang H, Evankovich J, Yan W, Nace G, Zhang L, Ross M, Liao X, Billiar T, Xu J, Esmon CT, Tsung A (2011). Endogenous histones function as alarmins in sterile inflammatory liver injury through Toll-like receptor 9 in mice. Hepatology.

[70] Shi Y, Evans JE, Rock KL (2003). Molecular identification of a danger signal that alerts the immune system to dying cells. Nature.

[71] Sperling C, Fischer M, Maitz MF, Werner C (2009). Blood coagulation on biomaterials requires the combination of distinct activation processes. Biomaterials.

[72] Nilsson B, Ekdahl KN, Mollnes TE, Lambris JD (2007). The role of complement in biomaterial-induced inflammation. Mol Immunol.

[73] Sarma JV, Ward PA (2011). The complement system. Cell Tissue Res.

[74] Zdolsek J, Eaton JW, Tang L (2007). Histamine release and fibrinogen adsorption mediate acute inflammatory responses to biomaterial implants in humans. J Transl Med.

[75] Abraham SN, St John AL (2010). Mast cell-orchestrated immunity to pathogens. Nat Rev Immunol.

[76] Anderson JM (2001). Biological responses to materials. Annu Rev Mater Res.

[77] Higgins DM, Basaraba RJ, Hohnbaum AC, Lee EJ, Grainger DW, Gonzalez-Juarrero M (2009). Localized immunosuppressive environment in the foreign body response to implanted biomaterials. Am J Pathol.

[78] McNally AK, Anderson JM (1994). Complement C3 participation in monocyte adhesion to different surfaces. Proc Natl Acad Sci U S A.

[79] Yang D, Jones KS (2009). Effect of alginate on innate immune activation of macrophages. J Biomed Mater Res A.

[80] Brodbeck WG, Colton E, Anderson JM (2003). Effects of adsorbed heat labile serum proteins and fibrinogen on adhesion and apoptosis of monocytes/macrophages on biomaterials. J Mater Sci Mater Med.

[81] Love RJ, Jones KS (2013). The recognition of biomaterials: pattern recognition of medical polymers and their adsorbed biomolecules. J Biomed Mater Res A.

[82] Hamad OA, Ekdahl KN, Nilsson B (2012). Non-proteolytically activated C3 promotes binding of activated platelets and platelet-derived microparticles to leukocytes via CD11b/CD18. Immunology.

[83] Rodriguez A, Meyerson H, Anderson JM (2009). Quantitative in vivo cytokine analysis at synthetic biomaterial implant sites. J Biomed Mater Res A.

[84] Jones JA, Chang DT, Meyerson H, Colton E, Kwon IK, Matsuda T, Anderson JM (2007). Proteomic analysis and quantification of cytokines and chemokines from biomaterial surface-adherent macrophages and foreign body giant cells..

[85] Mesure L, De Visscher G, Vranken I, Lebacq A, Flameng W (2010). Gene expression study of monocytes/macrophages during early foreign body reaction and identification of potential precursors of myofibroblasts. PLoS One.

[86] Xia Z, Triffitt JT (2006). A review on macrophage responses to biomaterials. Biomed Mater.

[87] Sindrilaru A, Peters T, Wieschalka S, Baican C, Baican A, Peter H, Hainzl A, Schatz S, Qi Y, Schlecht A, Weiss JM, Wlaschek M, Sunderkötter C, Scharffetter-Kochanek K (2011). An unrestrained proinflammatory M1 macrophage population induced by iron impairs wound healing in humans and mice. J Clin Invest.

[88] MacLauchlan S, Skokos EA, Meznarich N, Zhu DH, Raoof S, Shipley JM, Senior RM, Bornstein P, Kyriakides TR (2009). Macrophage fusion, giant cell formation, and the foreign body response require matrix metalloproteinase 9. J Leukoc Biol.

[89] McNally AK, Anderson JM (2002). Beta1 and beta2 integrins mediate adhesion during macrophage fusion and multinucleated foreign body giant cell formation. Am J Pathol.

[90] Helming L, Gordon S (2009). Molecular mediators of macrophage fusion. Trends Cell Biol.

[91] Gretzer C, Emanuelsson L, Liljensten E, Thomsen P (2006). The inflammatory cell influx and cytokines changes during transition from acute inflammation to fibrous repair around implanted materials. J Biomater Sci Polym Ed.

[92] Lynn AD, Kyriakides TR, Bryant SJ (2010). Characterization of the in vitro macrophage response and in vivo host response to poly(ethylene glycol)-based hydrogels. J Biomed Mater Res A.

[93] Santerre JP, Woodhouse K, Laroche G, Labow RS (2005). Understanding the biodegradation of polyurethanes: from classical implants to tissue engineering materials. Biomaterials.

[94] Sorokin L (2010). The impact of the extracellular matrix on inflammation. Nat Rev Immunol.

[95] Savill J, Gregory C, Haslett C (2003). Eat Me or Die.

[96] Lech M, Anders H-J (1832). Macrophages and fibrosis: How resident and infiltrating mononuclear phagocytes orchestrate all phases of tissue injury and repair. Biochim Biophys Acta.

[97] Pesce JT, Ramalingam TR, Mentink-Kane MM, Wilson MS, El Kasmi KC, Smith AM, Thompson RW, Cheever AW, Murray PJ, Wynn TA (2009). Arginase-1-expressing macrophages suppress Th2 cytokine-driven inflammation and fibrosis. PLoS Pathog.

[98] Diegelmann RF, Evans MC (2004). Wound healing: an overview of acute, fibrotic and delayed healing. Front Biosci.

[99] Hamilton JA (2003). Nondisposable materials, chronic inflammation, and adjuvant action.

[100] Vidal B, Serrano AL, Tjwa M, Suelves M, Ardite E, De Mori R, Baeza-Raja B, Martínez de Lagrán M, Lafuste P, Ruiz-Bonilla V, Jardí M, Gherardi R, Christov C, Dierssen M, Carmeliet P, Degen JL, Dewerchin M, Muñoz-Cánoves P (2008). Fibrinogen drives dystrophic muscle fibrosis via a TGFbeta/alternative macrophage activation pathway. Genes Dev.

[101] Wang Y, Wang YP, Zheng G, Lee VWS, Ouyang L, Chang DHH, Mahajan D, Coombs J, Wang YM, Alexander SI, Harris DCH (2007). Ex vivo programmed macrophages ameliorate experimental chronic inflammatory renal disease. Kidney Int.

[102] Lang R, Patel D, Morris JJ, Rutschman RL, Murray PJ (2002). Shaping gene expression in activated and resting primary macrophages by IL-10. J Immunol.

[103] Saraiva M, O’Garra A (2010). The regulation of IL-10 production by immune cells. Nat Rev Immunol.

[104] Murray PJ, Wynn TA (2011). Obstacles and opportunities for understanding macrophage polarization. J Leukoc Biol.

[105] Brys L, Beschin A, Raes G, Ghassabeh GH, Noel W, Brandt J, Brombacher F, Baetselier PD (2005). Reactive oxygen species and 12/15-lipoxygenase contribute to the antiproliferative capacity of alternatively activated myeloid cells elicited during helminth infection. J Immunol.

[106] Le SJ, Gongora M, Zhang B, Grimmond S, Campbell GR, Campbell JH, Rolfe BE (2010). Gene expression profile of the fibrotic response in the peritoneal cavity. Differentiation.

[107] Gurtner GC, Werner S, Barrandon Y, Longaker MT (2008). Wound repair and regeneration. Nature.

[108] Martin P, Leibovich SJ (2005). Inflammatory cells during wound repair: the good, the bad and the ugly. Trends Cell Biol.

[109] Eming SA, Krieg T, Davidson JM (2007). Inflammation in wound repair: molecular and cellular mechanisms. J Invest Dermatol.

[110] Sarrazy V, Billet F, Micallef L, Coulomb B, Desmoulière A (2011). Mechanisms of pathological scarring: role of myofibroblasts and current developments. Wound repair Regen.

[111] Hinz B (2007). Formation and function of the myofibroblast during tissue repair. J Invest Dermatol.

[112] Wynn TA (2008). Cellular and molecular mechanisms of fibrosis. J Pathol.

[113] Ninomiya K, Takahashi A, Fujioka Y, Ishikawa Y, Yokoyama M (2006). Transforming growth factor-beta signaling enhances transdifferentiation of macrophages into smooth muscle-like cells. Hypertens Res.

[114] Jabs A, Moncada GA, Nichols CE, Waller EK, Wilcox JN (2005). Peripheral blood mononuclear cells acquire myofibroblast characteristics in granulation tissue. J Vasc Res.

[115] Mooney JE, Rolfe BE, Osborne GW, Sester DP, van Rooijen N, Campbell GR, Hume DA, Campbell JH (2010). Cellular plasticity of inflammatory myeloid cells in the peritoneal foreign body response. Am J Pathol.

[116] Hutchison N, Fligny C, Duffield JS (1832). Resident mesenchymal cells and fibrosis. Biochim Biophys Acta.

[117] Tomasek JJ, Gabbiani G, Hinz B, Chaponnier C, Brown RA (2002). Myofibroblasts and mechano-regulation of connective tissue remodelling. Nat Rev Mol Cell Biol.

[118] Hinz B, Celetta G, Tomasek JJ, Gabbiani G, Chaponnier C (2001). Alpha-smooth muscle actin expression upregulates fibroblast contractile activity. Mol Biol Cell.

[119] Kenneth Ward W (2008). A review of the foreign-body response to subcutaneously-implanted devices: the role of macrophages and cytokines in biofouling and fibrosis. J Diabetes Sci Technol.

[120] Sharkawy AA, Klitzman B, Truskey GA, Reichert WM (1997). Engineering the tissue which encapsulates subcutaneous implants. I. Diffusion properties. J Biomed Mater Res.

[121] Saino E, Focarete ML, Gualandi C, Emanuele E, Cornaglia AI, Imbriani M, Visai L (2011). Effect of electrospun fiber diameter and alignment on macrophage activation and secretion of proinflammatory cytokines and chemokines. Biomacromolecules.

[122] Vasconcelos DP, Fonseca AC, Costa M, Amaral IF, Barbosa MA, Aguas AP, Barbosa JN (2013). Macrophage polarization following chitosan implantation. Biomaterials.

[123] Garg K, Pullen NA, Oskeritzian CA, Ryan JJ, Bowlin GL (2013). Macrophage functional polarization (M1/M2) in response to varying fiber and pore dimensions of electrospun scaffolds. Biomaterials.

[124] Bridges AW, Singh N, Burns KL, Babensee JE, Andrew Lyon L, García AJ (2008). Reduced acute inflammatory responses to microgel conformal coatings. Biomaterials.

[125] Zhang L, Cao Z, Bai T, Carr L, Ella-Menye J-R, Irvin C, Ratner BD, Jiang S (2013). Zwitterionic hydrogels implanted in mice resist the foreign-body reaction. Nat Biotechnol.

[126] Bryers JD, Giachelli CM, Ratner BD (2012). Engineering biomaterials to integrate and heal: the biocompatibility paradigm shifts. Biotechnol Bioeng.

[127] Beckstead BL, Tung JC, Liang KJ, Tavakkol Z, Usui ML, Olerud JE, Giachelli CM (2009). Methods to promote Notch signaling at the biomaterial interface and evaluation in a rafted organ culture model. J Biomed Mater Res A.

[128] Linnes MP, Ratner BD, Giachelli CM (2007). A fibrinogen-based precision microporous scaffold for tissue engineering. Biomaterials.

[129] Ratner BD (2011). The biocompatibility manifesto: biocompatibility for the twenty-first century. J Cardiovasc Transl Res.

[130] Mantovani A (2006). Macrophage diversity and polarization: in vivo veritas. Blood.

[131] Fukano Y, Usui ML, Underwood RA, Isenhath S, Marshall AJ, Hauch KD, Ratner BD, Olerud JE, Fleckman P (2010). Epidermal and dermal integration into sphere-templated porous poly(2-hydroxyethyl methacrylate) implants in mice. J Biomed Mater Res A.

[132] Bota PCS, Collie AMB, Puolakkainen P, Vernon RB, Sage EH, Ratner BD, Stayton PS (2010). Biomaterial topography alters healing in vivo and monocyte/macrophage activation in vitro. J Biomed Mater Res A.

[133] Bartneck M, Heffels K-H, Pan Y, Bovi M, Zwadlo-Klarwasser G, Groll J (2012). Inducing healing-like human primary macrophage phenotypes by 3D hydrogel coated nanofibres. Biomaterials.

[134] Bartneck M, Heffels K-H, Bovi M, Groll J, Zwadlo-Klarwasser G (2013). The role of substrate morphology for the cytokine release profile of immature human primary macrophages. Mater Sci Eng C Mater Biol Appl.

[135] Bartneck M, Schulte VA, Paul NE, Diez M, Lensen MC, Zwadlo-Klarwasser G (2010). Induction of specific macrophage subtypes by defined micro-patterned structures. Acta Biomater.

[136] Paul NE, Skazik C, Harwardt M, Bartneck M, Denecke B, Klee D, Salber J, Zwadlo-Klarwasser G (2008). Topographical control of human macrophages by a regularly microstructured polyvinylidene fluoride surface. Biomaterials.

[137] Martínez E, Engel E, Planell JA, Samitier J (2009). Effects of artificial micro- and nano-structured surfaces on cell behaviour. Ann Anat.

[138] Yim EKF, Leong KW (2005). Significance of synthetic nanostructures in dictating cellular response. Nanomedicine.

[139] Lord MS, Foss M, Besenbacher F (2010). Influence of nanoscale surface topography on protein adsorption and cellular response. Nano Today.

[140] Chen S, Jones JA, Xu Y, Low H-Y, Anderson JM, Leong KW (2010). Characterization of topographical effects on macrophage behavior in a foreign body response model. Biomaterials.

[141] Cao H, McHugh K, Chew SY, Anderson JM (2010). The topographical effect of electrospun nanofibrous scaffolds on the in vivo and in vitro foreign body reaction. J Biomed Mater Res A.

[142] Barbosa JN, Amaral IF, Aguas AP, Barbosa MA (2010). Evaluation of the effect of the degree of acetylation on the inflammatory response to 3D porous chitosan scaffolds. J Biomed Mater Res A.

[143] Spiller KL, Anfang RR, Spiller KJ, Ng J, Nakazawa KR, Daulton JW, Vunjak-Novakovic G (2014). The role of macrophage phenotype in vascularization of tissue engineering scaffolds. Biomaterials.

