# Supplementary figures and images for: The significance of macrophage phenotype in cancer and biomaterials
Source: Clin Transl Med. 2014 Nov 25;3:62. doi: 10.1186/s40169-014-0041-2 (PMC4884036; doi:10.1186/s40169-014-0041-2)

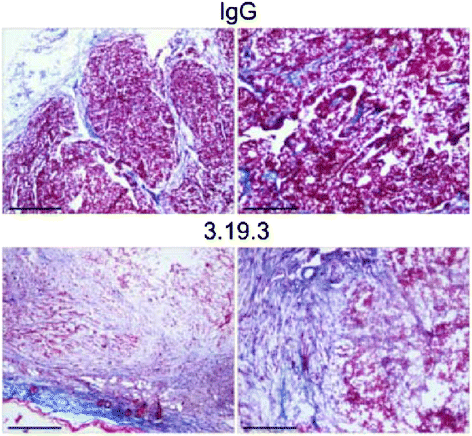

Supplement: Supplementary file 1 — Authors’ original file for figure 1 [file 40169_2014_41_MOESM1_ESM.gif]

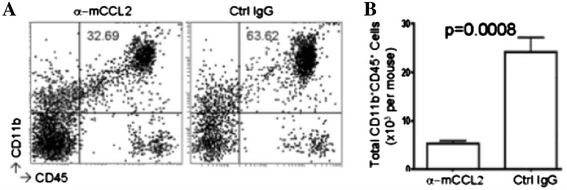

Supplement: Supplementary file 2 — Authors’ original file for figure 2 [file 40169_2014_41_MOESM2_ESM.gif]

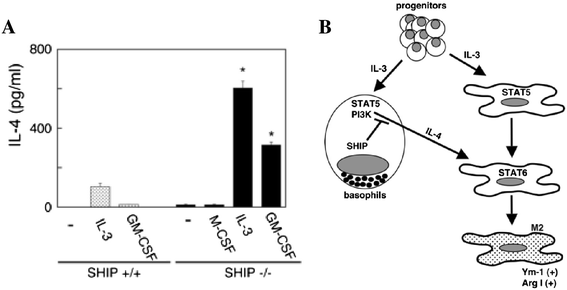

Supplement: Supplementary file 3 — Authors’ original file for figure 3 [file 40169_2014_41_MOESM3_ESM.gif]

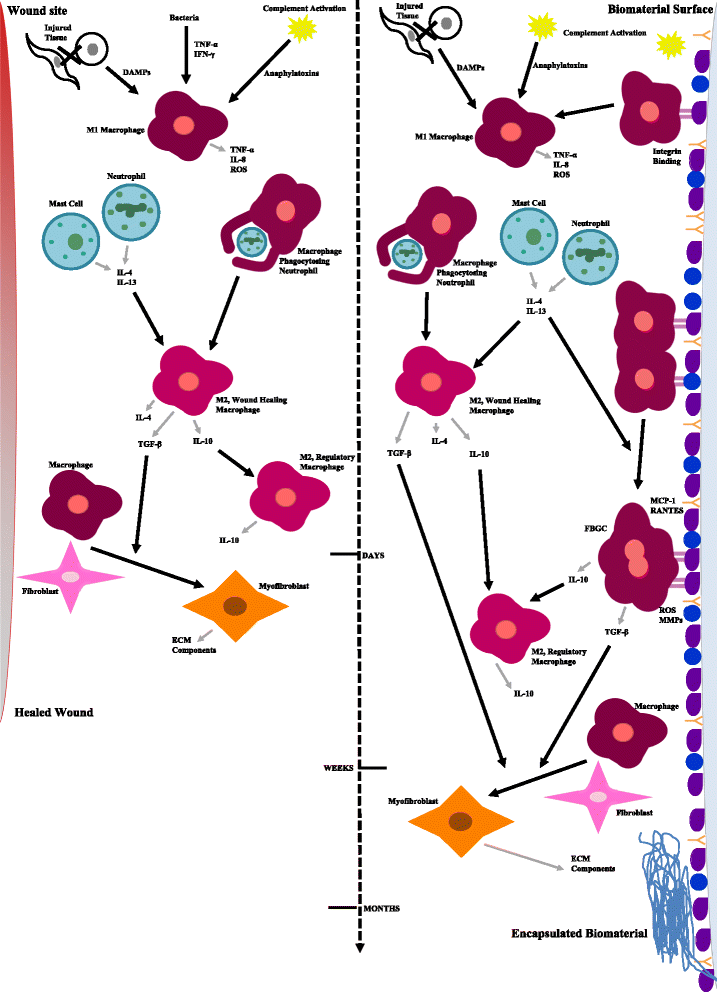

Supplement: Supplementary file 4 — Authors’ original file for figure 4 [file 40169_2014_41_MOESM4_ESM.gif]
