# Supplementary material for: The significance of macrophage phenotype in cancer and biomaterials
Source: Clin Transl Med. 2014 Nov 25;3:62. doi: 10.1186/s40169-014-0041-2 (PMC4884036; doi:10.1186/s40169-014-0041-2)
Supplement: Supplementary file 5 — Authors’ original file for figure 5 [file 40169_2014_41_MOESM5_ESM.pdf]

Table 1. Advantages and disadvantages of anti-cancer therapies targeting macrophage behaviors.

Approach	Advantages	Disadvantages
Anti-angiogenic therapy	Inhibit tumor growth and prevent metastasis [137, 138], improves efficacy of chemotherapeutics[33]	Must be used in combination with chemotherapeutics[34]; systemic effects[34, 139]
Recruitment inhibition	Prevent macrophages from entering tumor, becoming TAMs[140, 141]	Systemic effects[140]
Macrophage reprogramming	Macrophages secrete tumoricidal molecules [52, 142]	Local delivery necessary to avoid altering systemic Th1/Th2 paradigm[143]
