# Supplementary material for: The significance of macrophage phenotype in cancer and biomaterials
Source: Clin Transl Med. 2014 Nov 25;3:62. doi: 10.1186/s40169-014-0041-2 (PMC4884036; doi:10.1186/s40169-014-0041-2)
Supplement: Supplementary file 6 — Authors’ original file for figure 6 [file 40169_2014_41_MOESM6_ESM.pdf]

Table 2. Biomaterial influence on macrophage phenotype.

**Biomaterial Property**	**Macrophage Response**
Large fibers and pores (PDO)	M2 response, wound healing, angiogenesis [112]
Fiber size	
~0.6 μm (PLLA)	Minimal M1 activation, low FBGC population [110]
~1.6 μm (PLLA)	High FBGC population [110]
Hydrogels with pores (30-40 μm) (pHEMA-co-MAA)	M2 dominated, maximum vascularization, minimum fibrotic response [120]
Microgel coating (pNIPAm-co-PEGDA)	Reduction of M1 activation and cytokine secretion [113]
Zwitterionic hydrogels	Anti-inflammatory, pro-healing M2 macrophages, angiogenesis, no fibrous capsule [114]
Subintestinal submucosa	
Crosslinked with carbodiimide	M1 bias, chronic inflammation, prolonged healing [24]
Non-crosslinked	M2 bias, constructive remodeling [24]
Acetylated chitosan	
5% acetylated	Predominately M2, reduced fibrous capsule [111, 122]
15% acetylated	Presence of M1 macrophages [111, 122]
Glutaraldehyde crosslinked collagen	M1/M2 balance, improved vascularization [123]
Biologically-derived scaffolds	
Porcine submucosa, urinary bladder	M2, timely constructive tissue remodeling [23]
Human, porcine dermis	M1, prolonged healing [23]
